# Does Universal Masking Policy Prevent Hospital-onset COVID-19 among Patients in a Children’s Hospital?

**DOI:** 10.1017/ash.2025.379

**Published:** 2025-09-24

**Authors:** Kenta Kuruma, Ayumi Tada, Hanako Funakoshi, Meiwa Shibata, Yuho Horikoshi

**Affiliations:** 1 Tokyo metropolitan children’s medical center; 2 Department of Infectious Diseases, Medical Mycology Research Center, Chiba University

## Abstract

**Background:** Following the COVID-19 pandemic, various infection prevention strategies were implemented globally. Due to the challenges of asymptomatic transmission during the presymptomatic period, healthcare facilities in Japan adopted a strict universal masking policy for hospital staff and visitors. However, with the widespread availability of vaccines and therapeutic agents, as well as the reduced pathogenicity of the Omicron variant, particularly in younger populations, the universal masking policy was lifted for patients and visitors at our institution. This study evaluates the incidence of hospital-onset COVID-19 cases in a children’s hospital before and after this policy change. **Methods:** A retrospective analysis was conducted to assess hospital-onset COVID-19 cases among hospitalized patients at Tokyo Metropolitan Children’s Medical Center, Japan, between March 2020 and December 2024. Hospital-onset infection was defined as symptomatic COVID-19 diagnosed on or after the fifth day of hospitalization with a positive SARS-CoV-2 PCR result. Cases with a Ct value of ≥30, indicative of past infection, were excluded. The infection rate was calculated as the number of hospital-onset COVID-19 cases per 10,000 patient-days. During the universal masking policy period, all adults and children capable of wearing masks were required to wear them at all times in public spaces. From July 2023, patients and visitors were no longer required to wear masks unless otherwise indicated, while hospital staff continued universal masking during patient care but were allowed to remove masks for non-patient-care activities. The study compared two periods: the universal masking policy period (March 2020–June 2023) and the post-universal masking policy period (July 2023–December 2024). **Results:** During the universal masking policy period, there were 8 hospital-onset COVID-19 cases, compared to 10 cases in the post-universal masking policy period. The median ages of the patients were 106 months (IQR: 48-132) and 94 months (IQR: 56-152), respectively. The hospital-onset COVID-19 infection rates were 0.19 and 0.52 per 10,000 patient-days, respectively (p=0.07). None of the hospital-onset COVID-19 cases progressed to severe disease. **Conclusion:** The universal masking policy may have contributed to reducing the incidence of hospital-onset COVID-19 among patients and visitors in our children’s hospital to some extent. Further studies are needed to evaluate the long-term impact of masking policies on infection prevention in pediatric settings.

